# News and Events of the Week

**Published:** 1910-07-23

**Authors:** 


### News and Events of the Week.

The King has been pleased to approve the appointment of Dr. Hamilton Clelland Marr, M.D., as Medical Commissioner in Lunacy for Scotland, in place of Dr. John Fraser, who has retired on attaining the age limit.

The Chancellor of London University (Lord Rosebery) has appointed Dr. H. G. Cook, M.D., B.S., to be his Representative on the Court of Governors of the University College of South Wales and Monmouthshire.

Dr. Alonzo Henry Stocker, M.D., of Craigwell, Aldwick, Bognor, Sussex, and of Peckham House Asylum, Peckham, S.E., who died on April 24 last, at the age of eighty, left estate valued at £123,993 gross and £95,969 net.

The Secretary of the Hospital for Consumption and Diseases of the Chest, Brompton, invites anyone who may be interested in the treatment of consumptive cases by the system of graduated exercises in force at the sanatorium at Frimley, Surrey, to visit that institution this afternoon (Saturday), when the annual garden party will take place.

Dr. Dawson Williams, the editor of the *British Medical Journal*, met with a serious motor-car accident, from the result of which he is now lying at the Shelley Arms, Horsham, Sussex. While motoring on the main road from Guildford to Horsham his car collided with another car. Dr. Williams's condition, we understand, is improving.

Sir John Tweedy has given a present of books to the library of the Royal College of Surgeons, consisting of some rare editions of the fifteenth century, among which may be mentioned the Organon of Aristotle printed in Greek by Aldus, of Venice, 1495; the Consilia Cermisoni, Venice (Bonetus Locatellus), 1496; and "Summule in Libros Physicorum," Bologna, 1494.

At the Royal College of Surgeons the following professors and lecturers have been elected for the ensuing collegiate year: Hunterian Professors—Mr. Arthur Keith, Mr. F. Edridge-Greene, Mr. W. d'E. Emery, Mr. K. W. Goadby, and Mr. B. Moore; Arris and Gale Lecturer—Mr. G. Elliot Smith; Erasmus Wilson Lecturer—Mr. S. G. Shattock; Arnott Demonstrator—Mr. Arthur Keith.

Mr. Robert Mills, licentiate in midwifery, who was born at Bramfield on July 10, 1810, celebrated his 100th birthday last Sunday. He has received since, through the Rev. W. H. E. R. Jervis, rector of Orford, Suffolk, the following letter from Sir Arthur Bigge: "It having been brought to the King's notice that Mr. Robert Mills, a parishioner of yours, will attain the age of 100 years on Sunday, the 10th inst., I am commanded to ask you to be good enough to convey to him the expression of his Majesty's sincere congratulations on his having reached this grand old age. The King is interested to hear of all the good that Mr. Mills has rendered to his fellow-creatures during his long life, and trusts that he is in good health and that he may spend a happy birthday. Mr. Mills attended at a birth in his 92nd year, and beyond slightly defective hearing and eyesight he is in excellent health.

The Council of the University College of South Wales, Cardiff, has appointed Mr. E. E. Roberts, M.D., to the new Professorship of Pathology and Bacteriology. Mr. Roberts is a Bachelor of Medicine and Surgery of Manchester University and a Doctor of Medicine of Liverpool University. He gained his experience in clinical and *post-mortem* pathology and bacteriology in the Welsh Military Hospital during the South African war and at Liverpool and Bristol Universities.

Dr. C. G. Seligmann, M.D., who has recently conducted researches into the ethnology of the Veddas for the Government of India, and into the ethnology of the Sudan for the Government of those provinces, has been appointed to deliver at the London School of Economics during next session a systematic course of thirty lectures on ethnology, and to conduct a course of thirty seminar classes in connection therewith. He will receive the *status* of University Lecturer in Ethnology.

Messrs. Kegan Paul, Trench, Trubner and Co., Ltd., will publish shortly a work entitled "Medicine and the Church," being a series of studies on the relationship between the practice of Medicine and the Church's ministry to the sick, by Sir Clifford Allbutt, F.R.S., A. W. Robinson, D.D., Charles Buttar, M.D., Stephen Paget, F.R.C.S., the Bishop of Bloemfontein, Hon. Sydney Holland, Prebendary Fausset, M.A., Jane Walker, M.D., T. Hyslop, M.D., Ellis Roberts, M. Carta Sturge, H. G. Mackenzie, M.B. Edited with an introduction by Geoffrey Rhodes, with a Foreword by the Lord Bishop of Winchester. Crown 8vo. 6s. net.

On Wednesday, July 6, a collection of autograph letters and documents was sold at Sotheby's Rooms. A four-page letter of Miss Florence Nightingale, with its envelope, realised 12s. One lot was a letter from Dr. Edward Jenner to Dr. Barron, of Gloucester, dated September 24, 1824, in which he says: "In the course of the week I have been overwhelmed with voluminous letters on the subject of vaccination. The next number of *Duncan's Journal* will contain most of them. They are on the whole very pleasant, but here and there the effect of ignorance in conducting the vaccine process is most glaring." This, with two others, was sold for £4. At the same sale a very fine series of ninety letters from Charlotte Brontë to her friend Miss Ellen Nussey realised £102.

The following is the list of officers and other members of Council who have been elected at the annual general meeting of the Royal Society of Medicine for the society's year beginning October 1 next: President, Sir Henry Morris, Bt., M.B., F.R.C.S.; honorary treasurers, Sir William Selby Church, Bt., M.D., and Sir Francis H. Champneys, Bt., M.D.; honorary librarians, Mr. Rickman J. Godlee, M.S., and Dr. Norman Moore, M.D.; honorary secretaries, Dr. Arthur Latham, M.D., and Mr. Herbert S. Pendlebury, F.R.C.S. Other members of Council: Sir William Allchin, M.D., Dr. W. P. Herringham, M.D., Mr. H. T. Butlin, F.R.C.S., Mr. D'Arcy Power, M.B., F.R.C.S., Dr. R. A. Gibbons, M.D., Dr. H. D. Rolleston, M.D., Mr. J. Warrington Haward, F.R.C.S., Mr. Charters Symonds, M.S., and Mr. E. F. White, F.R.C.S.

His Majesty the King has graciously consented to continue to be an Honorary Fellow of the Royal College of Surgeons.

Dr. Addison has introduced a Bill into Parliament to provide for the compulsory instruction of hygiene and infant feeding in schools.

Sir William Treloar and his co-trustees have invited a number of members of the British Medical Association to visit and inspect the Lord Mayor Treloar's Cripples' Home and College at Alton Hants on Saturday, July 30. A special train will leave Waterloo at a quarter-past ten in the forenoon to take guests to the College, where luncheon will be served. The return train leaves Alton at half-past four in the afternoon.

Dr. Florence Nightingale Boyd, M.D., of Harley Street, London, W., sometime senior surgeon to the New Hospital for Women, and Vice-President and Hon. Treasurer of the Association of Registered Medical Women, who died on June 16 last, left estate valued at £19,008 19s. 7d. gross and £18,975 9s. 9d. net. She left various bequests to medical charities, which are recorded in our Gifts and Bequests.

The annual study-voyage of the Association Internationale d'Enseignement Médicale Complementaire (12 rue François Millet, Paris XVI.) will be to Belgium and the Netherlands, and will commence on August 8 next, members meeting at Lille. Ostend, Ghent, Brussels, Louvain, Antwerp, Rotterdam, The Hague, Utrecht, Leiden, Amsterdam, Liege, Spa, Luxembourg, and Strassburg will in turn be visited, and the various medical sights will be inspected at these centres. The inclusive cost of a member's ticket is 645 francs. Full particulars may be obtained on application to the secretary, at the above given address.

We have received from the secretary of the Docenten Verein für Aerztliche Ferienkurse (Berlin, Ziegelstrasse 10-11 Langenbeck Haus) a copy of the programme for the courses commencing in October next. It is one of the most interesting programmes the Association has as yet issued, and the subjects include practically the whole of special and general medicine, surgery, and the various departments, with practical courses. We can thoroughly recommend graduate students to try these courses, which in every way are ideal from a graduate point of view. A copy of the programme may be seen at the office of The Hospital, 29 Southampton Street, Strand, W.C., or may be obtained direct from the secretary, at the address given above.

The St. John Ambulance Association has issued details of a scheme for organising ambulance county companies to aid the wounded of the Territorial Force in time of war. These companies will be formed by the Territorial branch of the Association, and, in case of war in this country, their services will be offered to the War Office. County companies will be formed, on the general principle adopted in the past in forming St. John Ambulance Association classes of instruction in first aid, home nursing, etc. The members of a county company must be either registered medical practitioners, pharmacists, trained nurses, or persons who have obtained a St. John Ambulance Association certificate in first aid as regards men, and in first aid and home nursing as regards women. Every officer and member of a company, man or woman, will enter into an obligation to serve with the Territorial Force in his or her own county in case of war.

Messrs. Oetzmann and Co., Ltd., arranged a special view of their furnished cottages fitted with electric kitchen in operation and lighted by self-contained air-gas machines at the Japan-British Exhibition, Shepherd's Bush, on Wednesday last. The cottages are from the design of Mr. Henry White, F.R.I.B.A., and are exceedingly interesting in every respect. The firm issues a special illustrated booklet, which may be obtained on application to the offices, Hampstead Road, London, W.

We have received a copy of the new volume of *Truth's* "Queer Stories." Past issues of collections of these tales have been very popular, and this the latest addition to the series is in every way worthy of its predecessors. The stories have no particular medical interest, but are all interesting, while some are even thrilling. To those about to take their holidays we strongly recommend this excellent shilling's worth of reading. The volume can be bought at all bookstalls or may be had direct from the publishers, Cartaret Street, Queen Anne's Gate, S.W.

Special arrangements have been made by the Bath Corporation for the visit of members of the B.M.A. to the well-known hot springs city on Saturday, July 30 next. Invitations have been issued to 200 guests, who will leave by special train from Paddington at 10.40, and return to London by a train due at 8 p.m. No trouble has been spared to prepare an enjoyable and attractive programme for the visitors, who will be greatly aided in their excursions in the city by the interesting handbook to the hot springs of Bath compiled by Mr. Hatton, the director. This little guide-book, which is generously illustrated, gives full particulars about the history, merits, sights, sport, springs, and, indeed, all the attractions of the popular spa. There is, by the way, a special Bath exhibit (Stand 9) at the B.M.A. Exhibition at the Imperial Institute.

An anonymous donor has given £300 to the Dorset County Council Education Committee for an experiment to be made on an adequate scale in the treatment of the dental defects of children in the elementary schools of the county. It is proposed to extend the experiment over three months this year and three months next year. A whole-time dental surgeon is to be appointed at a salary of £78 for the three months, and is to visit as many schools as possible in the selected area—West Dorset. A charge of 6d. will be made to the parents of children treated. This experiment is of more than local interest, and differs from the usual hospital methods in that no gratuitous service will be asked of any doctor or dental surgeon, and it will not interfere with local practitioners, as it will take a class of work not previously dealt with, and it is hoped the habit of caring for the teeth once formed will lead to more practice for local practitioners in the future.

